# Assessment of the Prescribing Pharmacist’s Role in Supporting Access to Prescription-Only Medicines—Metadata Analysis in Poland

**DOI:** 10.3390/healthcare11243106

**Published:** 2023-12-05

**Authors:** Artur Owczarek, Dominik M. Marciniak, Rafał Jezior, Bożena Karolewicz

**Affiliations:** 1 Department of Drug Form Technology, Wroclaw Medical University, Borowska 211 A, 50-556 Wroclaw, Poland; dominik.marciniak@umw.edu.pl (D.M.M.); bozena.karolewicz@umw.edu.pl (B.K.); 2 Department of Data Processing Centers, Wroclaw IT Service Center, Namysłowska 8, 50-304 Wroclaw, Poland; rjezior@protonmail.com

**Keywords:** pharmacy prescription model, pharmacist prescribing practice, prescription-only medicines, pharmaceutical service

## Abstract

In 2020, pharmacists in Poland received additional authority to prescribe drugs. In this study, we analyzed prescribing after the implementation of this new responsibility. We assessed how the new regulation works in practice and what it means for the healthcare system in the area of access to prescription-only medicines. Data analysis included information on the prescriptions written, the type of substance according to the ATC classification, and data on the prescribing pharmacists. The study used over 2.994 million e-prescriptions written by pharmacists in Poland, which were made available by the e-Health Center. The largest group of drugs prescribed were drugs used in the treatment of cardiovascular diseases, accounting for 25% of all prescribed medications during the time of the analysis. The next prescription groups were for drugs used in gastrointestinal diseases and metabolic disorders, and those acting on the central nervous system, the respiratory system, and the musculoskeletal system. Among pharmaceutical prescriptions, 73% were pharmaceutical prescriptions issued in pharmacies at full price to the patient. The results indicate that pharmacists are eager to use their permission to prescribe drugs in authority situations. Almost three million records showed improved patient access to medicines in the healthcare system (approximately 5% of repeat prescriptions for all patients during the study period). These data confirm the possibility of cooperation between physicians and pharmacists in strengthening the efficiency of the patient healthcare system. An important conclusion from this work is the need to create the possibility for the pharmacist to access the information resources of the implemented Internet Patient Account system, including therapeutic indications for the drugs used.

## 1. Introduction

Regarding healthcare resources, the continued growth of healthcare needs and an aging population generate the need to analyze the situation and develop new solutions for supporting access to prescription-only medicines. Among the activities that health professionals perform is the prescribing of medicines within the scope of authority granted. Many countries are implementing solutions and practices related to prescribing by pharmacists [1]. It is necessary to conduct research in this area in order to objectively assess the impact of interventions in the area of pharmaceutical prescribing and to be able to identify directions for its development [2]. For example, in the United Kingdom and the United States, pharmacist prescribing has been practiced for nearly 20 years, with different models of prescribing adopted, such as independent, dependent, supplementary, and collaborative prescribing [3,4,5,6]. The most advanced type of prescribing practice is independent prescribing. In this model, the responsibility for the prescription of medicines belongs to pharmacists and is most similar to medical prescribing by physicians [7]. Dependent prescribing authority is given to a pharmacist after delegation by a physician [8]. Supplementary prescribing, which is a form of collaborative prescribing, is a drug therapy management model that was implemented in the United Kingdom in 2003 [9]. It is an optional partnership between an independent prescriber; a supplementary prescriber, for example, a nurse or pharmacist; and the patient, to implement an agreed patient-specific clinical management plan (CMP) [10]. Collaborative prescribing is a form of prescribing in which the primary responsibility for the safety of the pharmacotherapy rests with the physician [5,11]. Their tasks are to diagnose the patient and order the appropriate pharmacotherapy, while the pharmacist is responsible for monitoring the effectiveness of the therapy [12]. In Poland, a prescription written by a pharmacist can be issued for medicines with the “prescription” category of availability (Rx), excluding drugs containing narcotics and psychotropic substances [13]. As of 1 April 2020, pharmacists can issue a prescription to any patient in any health-threatening situation. In connection with this expansion of pharmacists’ prescribing permission in Poland, this study decided to evaluate the effects of pharmaceutical prescribing [14]. The types of prescribing carried out by pharmacists allowed in Poland that fall into the category of independent prescribing are listed in Table S1. Pharmacists prescribe drugs based on patient interviews, professional experience, and necessary medical knowledge. They make independent decisions, taking into account the current patient’s health state. The profession of a pharmacist is a profession of public trust. Pharmacists are subject not only to criminal and civil liability, but also to professional liability. Determining the reason and deciding whether to issue a pharmaceutical prescription depends on the independent competence of each pharmacist. Consequently, in the Polish healthcare system, no one can either order a pharmacist to issue pharmaceutical prescription or forbid it; it is an independent medical profession [14,15].

Any pharmacist with a license to practice also has the ability to issue prescriptions for his or her own needs (*pro auctore*) and prescriptions for immediate family members or persons in cohabitation (*pro familiae*). Those with the right to *pro familiae prescriptions* are spouses, persons in cohabitation, relatives or affinities in the pharmacist’s direct line, and those in the collateral line up to the degree of consanguinity between children of the siblings of the pharmacist. These prescriptions are issued in electronic form using a dedicated information system for professional healthcare professionals, the online platform gabinet.gov.pl. It is possible to issue a prescription in traditional paper form when online systems are out of order. Regardless of the form of the prescription issued by the pharmacist, each pharmacist is required to keep records of pharmaceutical prescriptions issued and a list of *pro auctore* and *pro familiae* prescriptions. Pharmaceutical prescriptions issued are recorded at the pharmacy or pharmacy point of sale for 5 years, while prescriptions issued for personal or family use must be recorded by the pharmacist in a personal register. If an electronic prescription is used, the pharmacist can record an amount of a drug product sufficient for 180 days of therapy. A paper prescription can be issued by a pharmacist for 120 days of treatment according to the dosage regimen of the drug product. At the same time, the amount of a contraceptive drug product on a paper pharmacy prescription cannot exceed 60 days of use, and the drug should only be prescribed as a continuation of a doctor’s order. In addition, as in the case of nursing prescribing, a pharmacist may prescribe up to four paper prescriptions for consecutive periods of use, not exceeding the aforementioned 120 days in total [14].

Pharmaceutical prescriptions for community pharmacy patients are filled only with full payment (not reimbursed) with an indication of the reason for dispensing the drug. Prescriptions issued for the pharmacist’s family, with the reason entered, should confirm the legitimacy of issuing the drug, falling within the professional powers of the pharmacist, and their reimbursement is possible [14,15]. Safe and effective prescribing, regardless of whether it is performed by a physician or representatives of other authorized professions, is an essential element of the healthcare system to ensure patient safety and high-quality patient care [16]. The aim of this work was to assess the scale of pharmacists prescribing medicines and investigate the pharmacist’s role in improving access to prescription-only medicines.

## 2. Materials and Methods

This study used anonymized digital data containing information on Rx prescribing carried out by pharmacists in the period from 1 May 2020 to 31 January 2022 in the territory of Poland, made available by the e-Health Center (CeZ), a government organization under the Ministry of Health responsible for the development of health IT systems in Poland. The data were made available and secured with an access password. The access password was shared with a designated member of the research team. The data file was password-protected against third-party interference, i.e., the risk of data modification, deletion, or unauthorized copying. The prescriptions of pharmacists with a valid license to practice their profession and registered in the register of pharmacists were analyzed. No exclusion criteria were applied, as all pharmacists with a professional license can issue prescriptions *pro auctore* and *pro familiae*. The nature of the data analyzed was the basis for defining a basic unit of calculation, called a prescription in this paper, which was then used in all the statistical analyses conducted. The term prescription means a single ATC code of a drug dispensed in a given month and year under a specific zip code by a pharmacist, defined by the gender, year of birth, and calculated age of the pharmacist. During the 21-month period analyzed, pharmacists in Poland wrote 2,994,746 prescriptions.

### Statistical Analysis

The basis for the statistical analyses conducted was a spreadsheet database obtained from the source data, where the columns contained the variables analyzed, and the subsequent rows contained individual ATC codes written out in a given month and year under a specific zip code by a pharmacist, defined by the pharmacist’s gender, year of birth, and age. Considering the number of prescription items written in a month, the number of rows of the experiment matrix of i = 2,330,106 translated into N = 2,994,746 individual ATC codes written at one time. The N value thus defined was treated as the final sample size of the study. A total of 13 variables were statistically analyzed:

1. One variable was the number of prescription items issued per month, measured on a quotient scale.
2. Four variables, including year of prescription, month of prescription, year of birth of the pharmacist, and age of the pharmacist, were on ordinal scales. Based on the year of the pharmacist’s birth, the age of the pharmacist was calculated, taking 2022 as the baseline. The pharmacist age calculated in this way was treated as a quotient variable. In part of the statistical analyses, the calculated quotient value of the age of the pharmacist was categorized and transposed to a nominal directional variable containing three age categories: up to 40 years; 41 to 60 years; over 60 years.
3. Five variables, including the type of prescription written, the voivodeship where the prescription was written, the postal code of the place where the prescription was written, the ATC code of the drug substance written, and the name of the drug substance, were nominal scale variables.
4. Three variables, including the gender of the pharmacist, form of the prescription written, and information on the form of payment (level of reimbursement), were nominally dichotomous.

A general characterization of the variables in the quotient scales was carried out by determining descriptive statistics for them: count, mean value, 95% significance interval for the mean value median, standard deviation, and spread. The normality of distribution was verified with Kolmogorov–Smirnov and Lilliefors tests, and homogeneity of variance was verified with Levene and Brown–Forsythe tests. All the quotient variables present in the analyzed database, at an assumed significance level of α = 0.05, did not meet the assumptions of normality of distribution and homogeneity of variance.

The general relationships and correlations between all analyzed variables included in the database, regardless of the scale that characterizes them, were initially evaluated using generalized principal component analysis (PCA). The constructed PCA model was estimated using the NIPALS iterative algorithm, the convergence criterion was set at 0.00001, and the maximum number of iterations was 100. The number of components was determined using the V-fold cross-check method by determining of the maximum predictive ability (Q^2^). The obtained optimal PCA model was reduced to 2 principal components and visualized on a PC 1 vs. PC 2 load chart, which allowed us to pre-select the variables that had the most significant impact on the variance in the analyzed experiment matrix. The correlations selected in this way were then subjected to further statistical analysis. The statistical significance of correlations between variables on both quotient and ordinal scales was assessed by calculating r-Pearson linear correlation matrices and nonparametric R-Spearman correlations. The statistical significance of the determined values of the r-Pearson and R-Spearman parameters was determined by a *t*-test assuming a level of α = 0.05. All calculated values of r and R were highly statistically significant. The degree of correlation of these variables was visualized on line graphs of functions of best fit to empirical data.

The statistical significance of the differences between the mean values determined for the quotient variables within the categories into which the nominal variables were divided was assessed. Due to the failure to meet the assumptions of normality of distribution and homogeneity of variance, we used the non-parametric Mann–Whitney U test for independent samples when two means were compared with each other, and the non-parametric Kruskal–Wallis analysis of variance when a nominal variable divided into more than two categories was assigned. Therefore, the basic statistical analysis that was used to assess the statistical significance of the degree of correlation between them was constructed multivariate tables of counts analyzed by the non-parametric chi^2^ test. In all statistical analyses, the level of significance was assumed to be equal: α = 0.05. Statistical analyses were carried out using the computer programs STATISTICA PL^®^ version 13.5 and R-Software^®^ version 4.0.3.

## 3. Results

**Figure 1:**
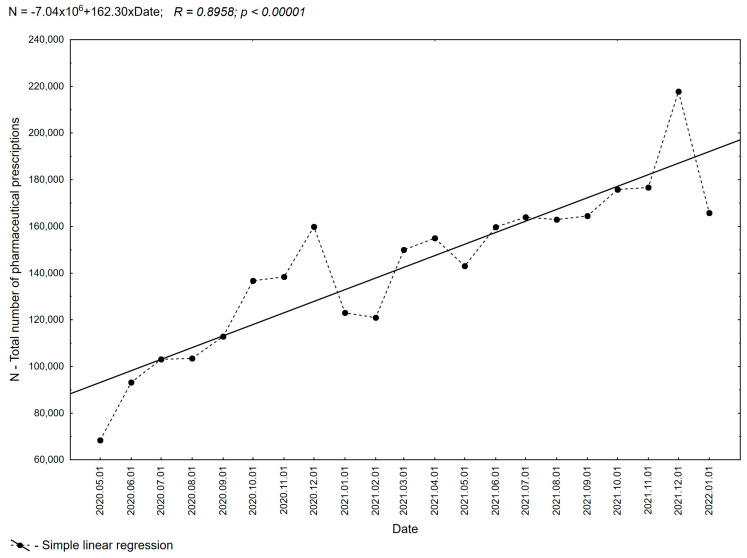
The total number of all pharmaceutical prescriptions in Poland, along with the trend line in subsequent months, for the period from 1 May 2020 to 31 January 2022.

An analysis of pharmaceutical prescribing in Poland makes it possible to determine to what extent pharmacists use their extended rights regarding pharmaceutical prescribing. The new laws were granted in April 2020, and then, from 15 May 2020, a government application was launched to enable electronic prescribing. In the studied period from 1 May 2020 to 31 January 2022, pharmacists in Poland issued 2,994,746 prescriptions, of which 2,187,275 (73.03%) were pharmaceutical prescriptions issued in pharmacies following the law for full payment from the patient (Table 1). The results of the analysis demonstrate that pharmacists use the permission they have been granted to prescribe drugs, as shown in Figure 1, which clearly shows an increase in the number of prescriptions in successive months of the 21-month period evaluated. The evaluation of the data shows that since the establishment of the regulation, the number of prescriptions issued has steadily increased from a value of 68,422 prescriptions issued in May 2020 to 217,763 prescriptions in December 2021 (an increase of more than 310%). The rest of the analyzed data (about 13.88%) consisted of prescriptions issued by pharmacists to family members and *pro auctore prescriptions* (13.08%).

**Figure 2:**
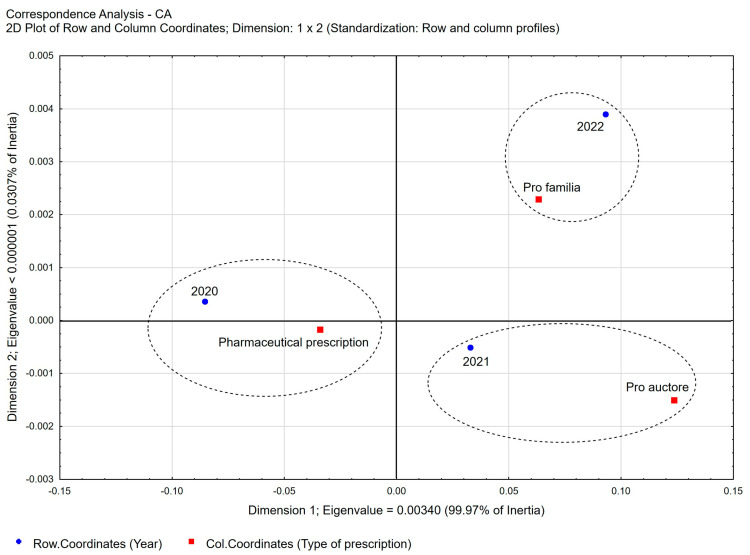
Correspondence Analysis (CA) for the following variables: type of pharmaceutical prescription vs. year. Two-dimensional plot of row and column coordinates—Dimension 1 vs. Dimension 2.

The vast majority of the prescriptions prescribed were covered by full payment (96.78%), despite the potential eligibility for reimbursement. Under the current state of the law in Poland, only *pro auctore* and *pro familiae prescriptions* can also be issued as reimbursable prescriptions. However, even for these prescriptions, pharmacists in Poland are cautious when deciding whether to indicate reimbursement on a prescription. The propensities to prescribe reimbursed prescriptions in the case of *pro familiae prescriptions* were 69.44% and 30.54% in the group of *pro auctore prescriptions*. A detailed analysis of the type of prescriptions written by pharmacists in each year of the study period shows a slight decrease in the percentage of pharmaceutical prescriptions in the total number of prescriptions (Figure 2) from 76.68% in 2020 to 71.64% in 2021 and 69.01% in 2022, respectively (Table 2). At the same time, in the total number of pharmaceutical prescriptions, the percentage share of *pro auctore prescriptions* increases from 10.71% in 2020 to 15.59% in 2022, and that of *pro familiae* prescriptions increases from 12.60% in 2020 to 15.40% in 2022, respectively.

The total number of pharmaceutical prescriptions written with reimbursement possible in the group of *pro auctore* and *pro familiae* prescriptions was 3.22% of all prescriptions (see Table 3). Analyzing the data in Table 4 on the share of pharmacist prescriptions with reimbursement by year, it is possible to observe a slight but statistically significant percentage increase in prescriptions written with full payment (from 96.52% in 2020 to 97.22% in 2022) and a decrease in prescriptions written with reimbursement (from 3.48% in 2020 to 2.78% in 2022).

**Figure 3:**
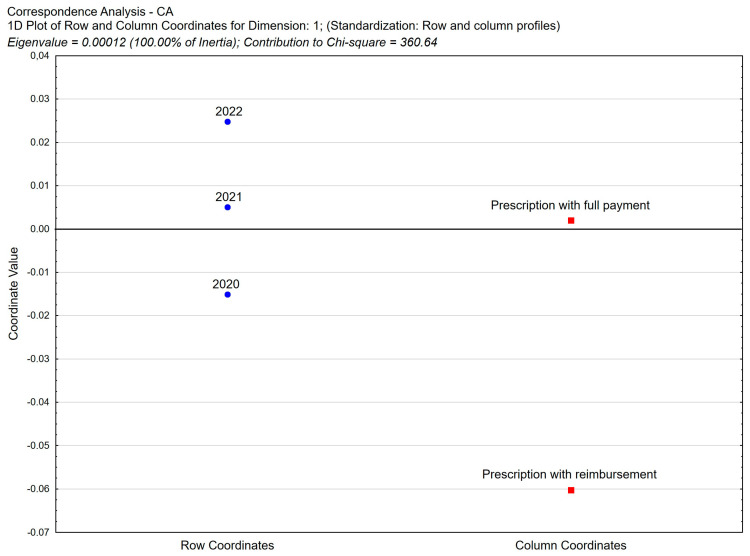
Correspondence analysis (CA) of the following variables: payment type vs. year. Two-dimensional plot of row and column coordinates—Dimension 1 vs. Dimension 2.

**Figure 4:**
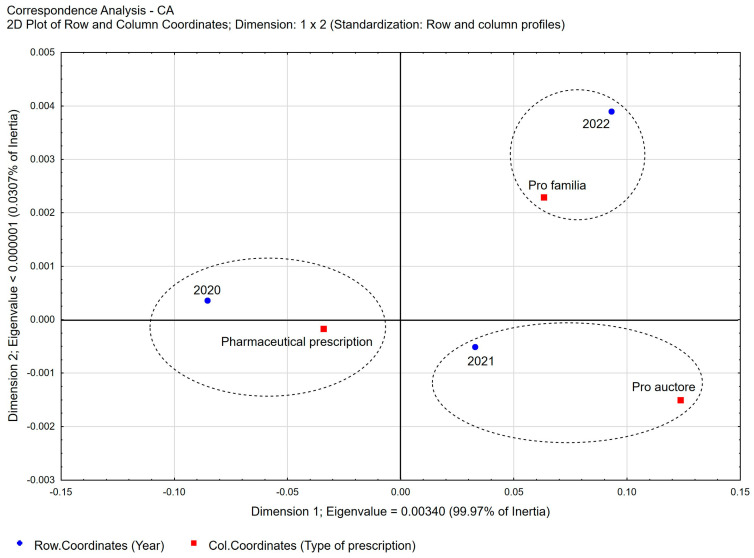
Correspondence analysis (CA) of the following variables: type of payment vs. year in the group of “*pro auctore*” and “*pro familiae*” prescriptions. Two-dimensional plot of row and column coordinates—Dimension 1 vs. Dimension 2.

According to our detailed analysis, the trend of changes in the share of pharmaceutical prescriptions with reimbursement in the groups of *pro auctore* and *pro familiae prescriptions* is maintained, with an increasing share of prescriptions written with full payment (Figure 3). In 2020, among *pro auctore* and *pro familiae prescriptions*, reimbursed prescriptions accounted for 9.23%. In the following years, this value decreased to 7.04% in 2021 and 5.77% in 2022 (Table 5, Figure 4).

In the Polish healthcare system, in addition to their dominant role of electronic prescriptions, it is permissible for a pharmacist to issue a prescription in paper form. This option can be used only in certain situations, including a lack of access to the pharmacy’s prescribing system (Medical Information System, SIM, Warszawa, Poland). The data show that this form of prescribing is used by pharmacists primarily to write *pro auctore* and *pro familiae* prescriptions following established law. Of the total prescriptions written by pharmacists, only 0.61% (Table 6) were paper prescriptions (18,293 prescriptions), of which more than 51.17% were *pro auctore prescriptions*, 37.99% *pro familiae prescriptions*, and only 10.85% pharmaceutical prescriptions (1984 prescriptions, see Table S2). The low share of paper prescriptions in all forms of pharmaceutical prescribing is mainly limited to *pro auctore* and *pro familiae prescriptions* (89.16%) and indicates the widespread use of e-prescribing while ensuring that pharmacists can write paper prescriptions following the established law.

**Figure 5:**
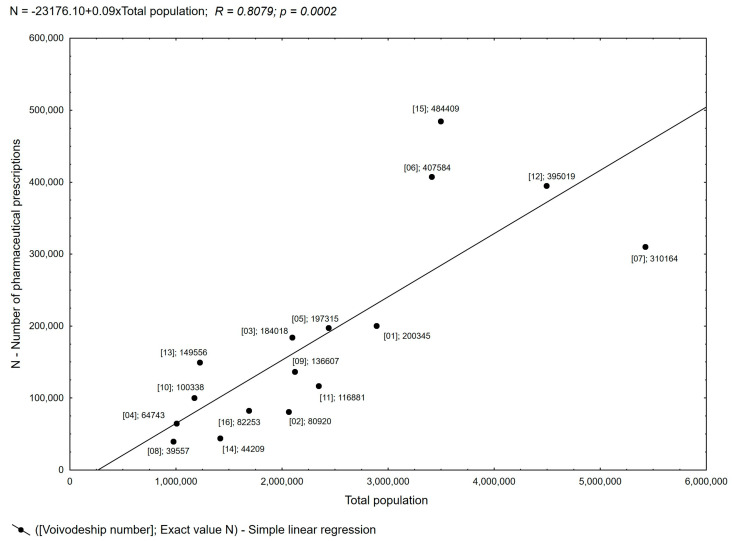
Pearson’s linear correlation between population and the number of pharmaceutical prescriptions in a voivodeship.

Administratively, the healthcare system in Poland covers 16 voivodeships. The largest number of prescriptions were filled in four voivodeships: Greater Poland (16.18%), Lesser Poland (13.61%), Silesia (13.19%), and Masovia (10.36%) (Table S3). Considering the Central Statistical Office’s 2021 data on the resident population, these are the voivodeships with the highest populations, at 3.496 million, 3.410 million, 4.492 million, and 5.425 million, respectively [17,18]. Practically throughout the country, a nearly linear relationship is observed between the number of pharmaceutical prescriptions and the population in a given area. Local deviations from this trend were observed for three voivodeships, Masovia, Greater Poland, and Lesser Poland, where the correlation between the number of pharmaceutical prescriptions and the population in the area was not fully confirmed, as shown in Figure 5. This analysis made it possible to show the issuing of pharmaceutical prescriptions at a similar level throughout Poland.

**Figure 6:**
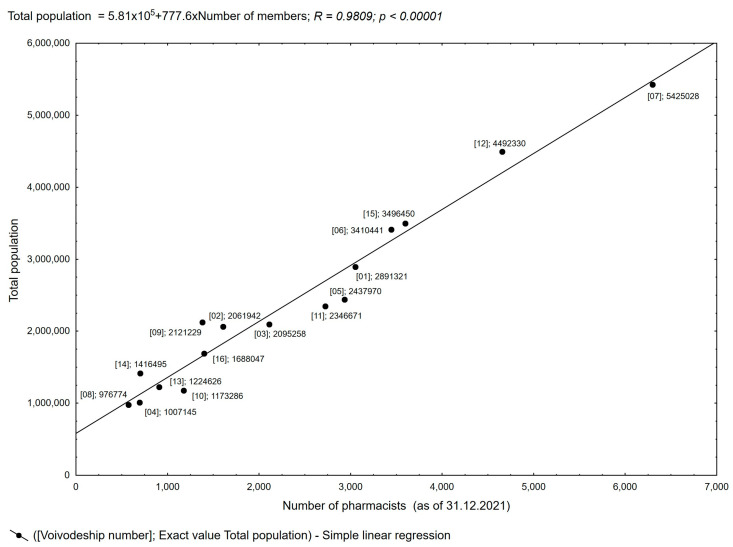
Pearson’s linear correlation between population and the number of pharmacists in a given voivodeship.

For the analysis of the practice of pharmaceutical prescribing, it is also important to correlate the size of the population residing in a given area with the number of pharmacists with the right to practice and, in turn, to correlate the number of prescriptions issued with the number of pharmacists (data as of 31 December 2021 obtained from the Pharmacy Chamber). The correlation of the population with the number of pharmacists in each voivodeship is positive and proportional throughout Poland, which guarantees patients equal access to pharmaceutical services. The data regarding the number of pharmaceutical prescriptions do not fully correlate with the number of pharmacists in a given area, as shown in Figure 6. The observed deviations in six voivodeships indicate a lower or higher number of pharmaceutical prescriptions per pharmacist concerning the stated average (Figure S1). These deviations confirm the flexibility and willingness of pharmacists to provide access to the pharmaceutical prescription service regardless of where the patient uses the service and the number of patients per pharmacist.

Among the most common groups of ATC-classified substances prescribed by pharmacists, drugs belonging to the following categories were identified: cardiovascular system (C), alimentary tract and metabolism (A), nervous system (N), respiratory system (R), musculoskeletal system (M), anti-infectives for systemic use (J), and dermatologicals (D). These accounted for more than 86% of all pharmaceutical prescriptions examined (Table 7). The most numerous drug substances written by pharmacists according to the Anatomical Therapeutic Chemical Classification (ATC) group code were drugs belonging to group C—which includes drugs for the treatment of cardiovascular diseases, accounting for 25.04% of all prescriptions written. In this group are drugs classified as C 07 AB—selective β-blockers (respectively, 3.16% C07 AB 07—bisoprolol, 1.79% C 07 AB 02—metoprolol, C 07 AB 12—nebivolol, 1.10% of all prescriptions in the analyzed period; see Table S4), C 09 AA 05 (a drug acting on the renin–angiotensin system from the group of angiotensin-converting enzyme inhibitors)—ramipril (1.69%), and C 10 AA 07 (a drug that reduces lipid concentrations from the group of HMG-CoA reductase inhibitors)—rosuvastatin (1.09%). Another large group of drugs prescribed by pharmacists were medicinal products classified according to the ATC classification as group A—drugs used in gastrointestinal diseases and metabolic disorders—and in total, they accounted for 13% of all pharmaceutical prescriptions. Among this group, A 10 BA 02 oral hypoglycemic drugs from the group of biguanide derivatives—metformin (1.54% of the total)—and A 02 BC 02 drugs used in peptic ulcer disease and reflux disease from the group of proton pump inhibitors—pantoprazole (1.47%)—predominated. Next, pharmacists prescribed drugs from the ATC groups N (8.72%), R (8.67%), M (8.66%), and J (8.37%) according to the Anatomical Therapeutic Chemical Classification, acting on the central nervous system, respiratory system, and musculoskeletal system, and drugs used in infections, respectively. In the group of drugs acting on the musculoskeletal system, and anti-inflammatory and anti-rheumatic drugs from the acetic acid derivatives group—M 01 AB 05—diclofenac accounted for 1.63% of all drugs prescribed during the period under review. Similarly, in the group of drugs used in infections, the most frequently prescribed drugs were antibacterial drugs for internal use from the macrolide group J 01 FA 10—azithromycin (1.47% of prescriptions). Analyzing the prescribing practices of pharmacists, numerous groups of prescriptive drugs were the following substances: H 03 AA 01—levothyroxine (2.99%), G 03 AA 12—drospirenone in combination with estrogen (1.12%), and S 01 CA 06—fludrocortisone in combination with anti-infectives (1.05%). 

In addition to licensed medicinal products, a group of pharmaceutical prescriptions involving magistral preparations was identified, accounting for about 1.99% of all prescriptions. A total of 35,927 prescriptions were filled during the analysis period. This is a new cognitive phenomenon, indicating that access to medications prepared at the pharmacy for individual patient needs is supported by the pharmacist’s prescribing. 

**Figure 7:**
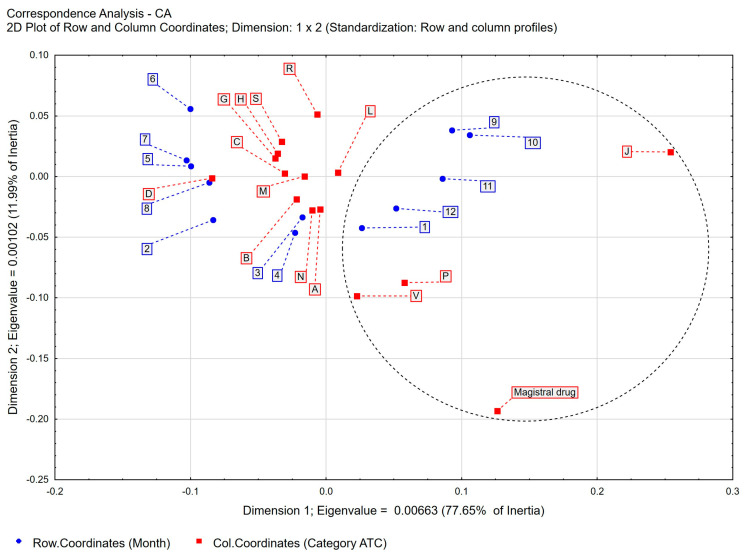
Correspondence analysis (CA) of the following variables: main ATC group vs. month of prescription. Two-dimensional plot of row and column coordinates—Dimension 1 vs. Dimension 2.

The most widely prescribed group of Class C drug substances covering drugs for the treatment of cardiovascular diseases occurred in all types of prescriptions written by pharmacists: pharmaceutical prescriptions, and *pro auctore*, and *pro familiae* prescriptions. The number of prescriptions prescribed throughout the study period varied by ATC classification, ranging from 749,799 prescriptions of substances with cardiovascular effects to 2725 prescriptions of substances in the various group. When analyzing the differences in prescribing by ATC classification code, drugs belonging to group P—substances with antiparasitic, insecticidal, and repellent effects—were prescribed more frequently in the *pro auctore prescribing* group than in the groups of other types of prescribing, accounting for 0.88% of total prescribing. When analyzing the frequency of groups prescribing active substances by ATC classification in *pro auctore* prescriptions, these were statistical significant from the other groups (Figure S2). The phenomena described above are of statistical significance in the group of *pro auctore* prescriptions; however, they do not represent numerous significant prescriptions, affecting the evaluation of the phenomenon as a whole. In the group of *pro familiae* prescriptions, respectively, pharmacists prescribed drugs from groups more often compared to pharmaceutical prescriptions: L—anticancer and immunomodulatory drugs, R—drugs acting on the respiratory system, N—drugs acting on the central nervous system, and J—drugs used in infections. Similarly, in the group of *pro familiae prescriptions*, the ATC groups prescribed most often were identified; however, they do not represent statistical significance of numerical prescriptions, affecting the evaluation of the phenomenon of total pharmaceutical prescriptions. Upon analyzing pharmaceutical prescriptions by ATC classification code by month during the study period, drugs belonging to the following groups were prescribed more frequently in the months of September through January: J—drugs used in infections, P—substances with antiparasitic, insecticidal, and repellent effects, V—various (varia), and magistral preparation—prescription drugs. This indicates that the prescription of drugs from the groups most frequently prescribed were at a similar level throughout the year (Figure 7).

The next issue analyzed was the characteristics of pharmacists who made prescriptions, taking into account the year of birth, age of the pharmacist in 2022 (Figure S3), and gender (Table 8 and Table S5). A total of 39.33% of all prescriptions were written by pharmacists under the age of 40, 46.44% by pharmacists between the ages of 41 and 60, and 14.23% by pharmacists over the age of 60. Among prescribing pharmacists, the percentage of women was 76.58% and men 23.42%. The average age of men was 43.4 years and of women 46.1 years.

**Figure 8:**
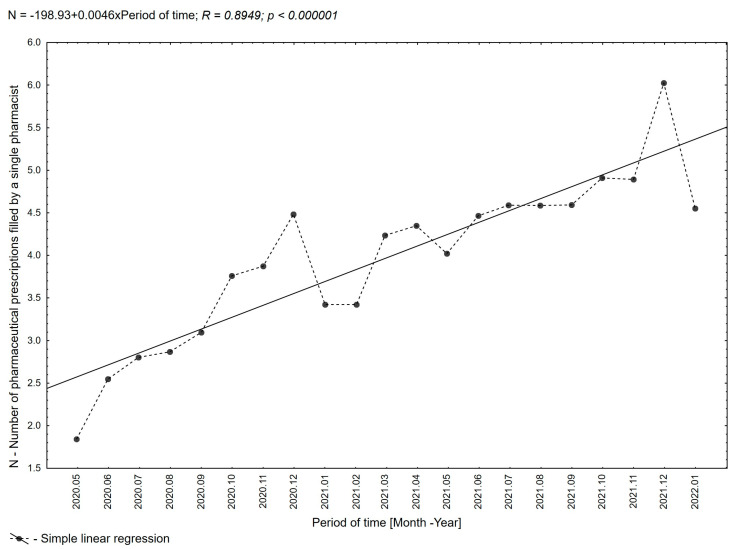
Trend line with the average number of pharmaceutical prescriptions filled by a single pharmacist in consecutive months of the period evaluated.

The average number of pharmaceutical prescriptions filled by a single pharmacist grew over the evaluated period from an average of 1.84 prescriptions in the first month of analysis in 2020 to an average of 6.02 prescriptions in December 2021 (Figure 8). 

**Figure 9:**
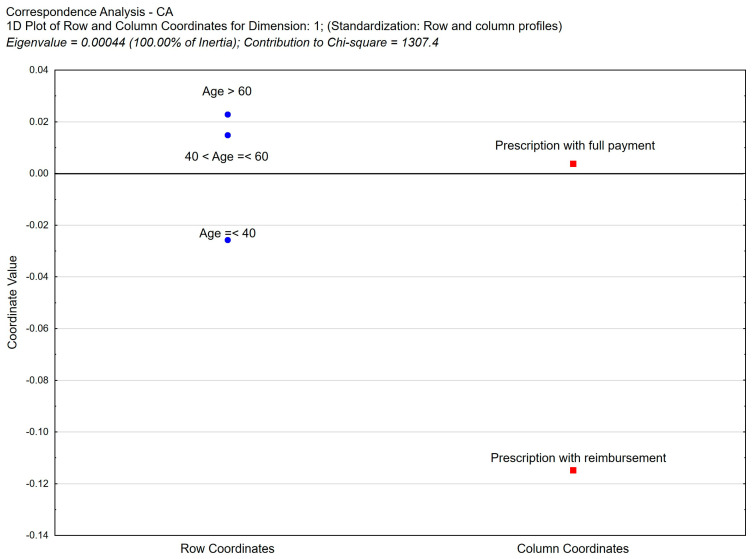
Correspondence analysis (CA) of the following variables: age of pharmacist vs. form of payment. Two-dimensional plot of row and column coordinates—Dimension 1 vs. Dimension 2.

In the analysis of pharmacist prescribing, there is a statistically significant correlation between the age of pharmacists writing prescriptions and their reimbursement. The highest percentage of prescriptions written with reimbursement (3.67%) is found in the group of the youngest pharmacists under the age of 40, and the percentage drops to 2.81% in the group of pharmacists over the age of 60 (Table S6, Figure 9).

**Figure 10:**
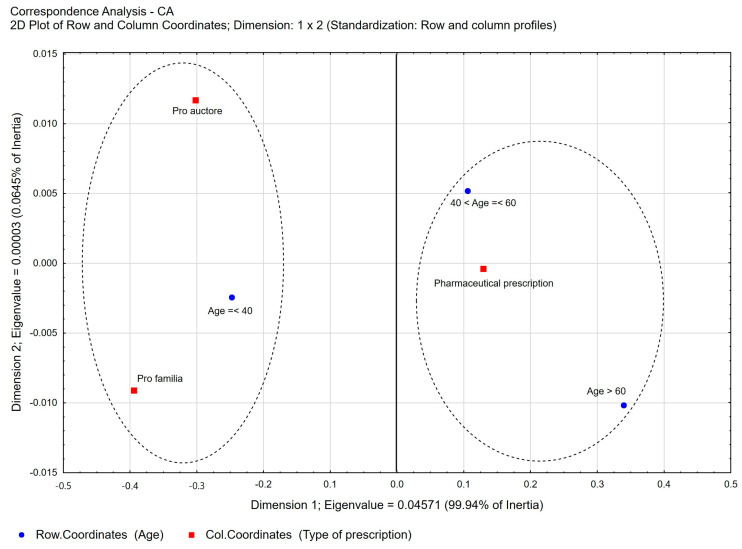
Correspondence analysis (CA) of the following variables: age of pharmacist vs. type of pharmaceutical prescription. Two-dimensional plot of row and column coordinates—Dimension 1 vs. Dimension 2.

A statistically significant correlation was also found between the age of pharmacists and the type of prescription. The participation percentage of pharmaceutical prescriptions increased with the age of the pharmacists (Figure 10). Among pharmacists under 40 years of age, it accounted for 62.12% of all prescriptions written, and increased to 77.67% among pharmacists aged 41 to 60 (Table S7). Among pharmacists over the age of 60, it was highest at 88.05%. *Pro auctore* and *pro familiae* prescriptions, compared to pharmacist prescriptions, were more often written by younger pharmacists—aged up to 40—and accounted for 17.60% and 20.27% of prescriptions filled in this age group, respectively. *Pro auctore* and *pro familiae* prescriptions among pharmacists aged 41–60 already accounted for 11.28% and 11.05%, respectively. 

## 4. Discussion

Pharmacy practice is evolving to improve patient care, and prescribing medications and educating on their proper use is one of the tools to ensure the delivery of safe and effective healthcare [19]. Limited access to expert healthcare professionals in the situation of an insufficient number of specialists with a simultaneously growing population of patients in need is one of the most important problems in today’s healthcare systems. Pharmacists, compared to other healthcare professionals, are more readily available, as noted in studies of patients in countries where pharmacist prescribing has been analyzed [20,21]. The PGEU indicates that pharmacies are the first line of advice, treatment, and referral for many people in Europe with common ailments, effectively preventing unnecessary emergency room visits. According to data published by the PGEU, 98% of patients can reach a pharmacy within 30 min, and 58% need only five minutes to do so [22]. In their work, Johnson et al. evaluated the cost-effectiveness of using pharmacists to prescribe medications, potentially positively relieving the burden on physicians in this regard [23,24]. Ongoing comparative studies have indicated that patients under the care of a prescribing pharmacist achieve better therapeutic outcomes, and physicians experience less work overload [25,26,27]. Currently, pharmacists are becoming largely responsible for helping patients navigate an increasingly complex and costly healthcare system, particularly regarding medications [27].

After analyzing the practice of pharmaceutical prescribing in Poland during the reviewed period, a clear trend was observed: the number of pharmaceutical prescriptions increased from 68,422 prescriptions issued in May 2020 to 217,763 prescriptions in December 2021. This steady rise following the introduction of expanded rights for pharmacists underscores a real need in this area. Interestingly, the vast majority of prescriptions written by pharmacists were filled with full payment (96.78%), despite the possibility of reimbursement for *pro auctore* and *pro familiae* prescriptions. This cautious approach may stem from concerns about challenging the legitimacy of reimbursement indications during audits. Pharmacists in Poland carefully consider whether to indicate reimbursement on a prescription and are cautious when deciding to indicate reimbursement on a prescription. The underlying reason for this phenomenon lies in the pharmacist’s lack of automatic access to the medical indication for drug use. Consequently, there is a potential risk of granting undue reimbursement. Unfortunately, this lack of access deprives the patient of the opportunity to benefit from reimbursement, thereby increasing their financial burden. Interestingly, a higher proportion of prescriptions issued with reimbursement occurs in the *pro familiae group*. This could be attributed to pharmacists’ awareness of existing authorizations for specific medical indications among family members, leading them to prescribe the drug for continued treatment. In such cases, pharmacists can refer to the patient’s entitlements related to the diagnosis during inspections. 

Of all prescriptions written by pharmacists, only 0.61% were prescriptions in traditional paper form. The small share of this form of pharmaceutical prescribing indicates the widespread use of e-prescribing while ensuring that pharmacists can write a paper prescription in the absence of SIM (Medical Information System) access. The observed near-linear relationship between the number of pharmaceutical prescriptions and the population in a given area indicates balanced access to the service. The results obtained from the prescriptions analysis reveal the actual extent of patients’ needs when requesting pharmaceutical prescriptions. Among the groups of ATC-classified substances prescribed by pharmacists in Poland, substances belonging to seven groups—in order of the highest number of prescriptions—include those targeting the cardiovascular system, alimentary tract and metabolism, nervous system, respiratory system, and musculoskeletal system, as well as anti-infectives for systemic use and dermatologicals. These groups accounted for more than 80% of pharmaceutical prescriptions. Similarly, drugs used to treat cardiovascular diseases constituted 25% of all prescriptions written. Similarly, a study in Wales conducted over a 10-year period (April 2011 to March 2021) analyzed prescribing by non-medical independent prescribers. They found that substances from seven groups of the BNF classification—cardiovascular, the nervous system, infections, the respiratory system, the endocrine system, the gastrointestinal system, and skin—accounted for about 80% of all prescriptions, with cardiovascular drugs being the dominant group [28,29]. These were also the groups of substances most frequently prescribed by pharmacists in our study. Additionally, it is worth noting the increase in the number of anti-infective drugs prescribed by pharmacists, who can contribute to the rational use of antimicrobial drugs [9].

This study analyzed the structure of human resources in terms of pharmaceutical prescribing. Currently, in Poland, the stock of pharmacists with an active right to practice registered in the register of pharmacists totals 37,261 people (data as of 31 December 2021, obtained from the Supreme Pharmaceutical Chamber). Among them, 26,008 work in 11,866 pharmacies (as of 2021). In 2021, there was an average of 2926 people per community pharmacy and pharmacy point, which is 29 more than in 2020. Additionally, in September 2021, pharmacies were visited by an average of 4370 patients, representing a 13.5% increase compared to the same period of 2020 [22]. Regarding physicians, Poland has 238 physicians per 100,000 people, which is one of the lower rates in Europe. In 2019, 291.5 million medical consultations were conducted, with 69.06 million related to repeat prescriptions. In terms of the number of pharmacists, according to the OECD report, the average number of pharmacists per 100,000 inhabitants was 86 in 2019. Additionally, there were 28 pharmacies per 100,000 citizens [30]. Poland’s figures do not significantly deviate from the European average in this regard. In 2020, there were 76 pharmacists and 33 pharmacies per 100,000 citizens [31,32]. The abundance of pharmacies and easy access to pharmacists are the primary reasons patients utilize pharmaceutical services [33]. Notably, Polish pharmacists enjoy very high public trust; in 2019, 90.4% of Poles declared that they trust pharmacists [34]. These factors contribute to the rapid popularity of the new pharmaceutical services being implemented. The inclusion of pharmacists in the prescribing process within the public healthcare system is an important element in enhancing access to prescription-only medicines and allows for the further development of their professional competence [35]. These perceived trends are substantiated by the data on the number of pharmaceutical prescriptions issued, as presented by the Supreme Pharmaceutical Chamber. In 2022, during the period from January to September, pharmacists issued 2,183,275 pharmaceutical prescriptions, compared to the same period in 2020, which saw 1,707,074 pharmaceutical prescriptions. This represents a significant 27.9% increase [16]. The limited participation in reimbursed prescribing highlights a need for the development of services that ensure patients’ rights to reimbursement for the cost of the prescription-only medicines they purchase. To address this, there should be clarity regarding the tool that confirms a patient’s right to drug reimbursement. Ideally, pharmacists should have access to the patient’s indications for the drug within the SIM (Medical Information System). Looking ahead, Poland aims to establish a continuing prescription service, wherein pharmacists can issue further reimbursed prescriptions for patients based on the doctor’s orders.

The widespread availability of pharmacies means that they are often the first point of contact for patients seeking consultation on pharmacotherapy or a health condition, as confirmed by the World Health Organization [36]. Community pharmacies have demonstrated their value to patients and health systems during the health crisis associated with the COVID-19 outbreak, among others. They are poised to further expand this contribution through sustainable collaboration with other healthcare professionals and providers in the community [37]. During the onset of the crisis, several countries implemented measures to ensure continuity of treatment for patients with chronic diseases and reduce unnecessary visits to primary care or hospitals. Pharmacists were given the opportunity to renew prescriptions for chronic-use medications in six European countries. Additionally, the electronic transfer of prescriptions to pharmacies was enabled in cases where this had not been implemented yet. Specifically, in Belgium, Croatia, Germany, the Netherlands, Portugal, and the United Kingdom, the scope of pharmacists was expanded to include alternatives for emerging drug shortages. In France, Ireland, and Portugal, pharmacists in pharmacies were able to renew and fill prescriptions for patients with certain chronic diseases. These initiatives highlight the crucial role of pharmacists in ensuring continuity of care and optimizing patient outcomes during challenging times [38]. In the UK, since 2006, legal changes have allowed pharmacists to prescribe independently (known as independent prescribing), wherein all responsibility for diagnosing the condition and prescribing appropriate pharmacotherapy rests with the pharmacist [39,40]. Pharmacists can qualify for this role after completing a pharmacist independent prescribing program accredited by the General Pharmaceutical Council. A review of the literature on the opinions of patients, physicians, and pharmacists after service implementation demonstrated a positive impact of complementary and independent prescribing on the clinical status of patients and healthcare [41,42,43]. Stakeholders working with prescribing-authorized pharmacists highlighted several benefits, including of ease of patient access to healthcare services, improved patient outcomes, improved pharmacist job satisfaction, and reduced physician workload [44,45]. 

Pharmaceutical prescription analysis is an important element for better forecasting of epidemiological trends, health behaviors, and health needs. The document “*The State Medicines Policy 2018-22*” focuses on the widespread use of pharmacy services and simple access to pharmacists working in them. It highlights the possibility of supporting the healthcare system by optimally utilizing the potential of this professional group [46]. In Poland, the number of repeat prescriptions in the healthcare system was 31.97 million in 2020 and 30.15 million and 2021, accounting for 21.92 and 18.90% of all medical prescriptions in primary care. The implementation of prescribing services will shift some visits related to repeat prescriptions from primary healthcare to pharmacies, enhancing access to prescription-only medicines after consultations with healthcare providers. The observed phenomenon confirms the effectiveness and usefulness of systemic pharmaceutical prescribing capability, leading to its widespread adoption across the country.

## 5. Conclusions

Including pharmacists in the implementation of health services is beneficial for patients and the growing needs of the healthcare system. Highly developed countries actively encourage pharmacists to make full use of their qualifications. The implemented pharmaceutical prescribing regulations in Poland undoubtedly increase access to necessary prescription-only medicines. By expanding the role of pharmacists in the healthcare system, it is possible to enhance the efficiency of healthcare services while relieving the burden on other health professions and promoting pharmaceutical care. The results of our analysis show that pharmacists in Poland responsibly utilize their expanded prescribing authority. However, further research is needed to identify possible barriers to developing this service for the benefit of patients. The obtained analysis will allow for the development of detailed recommendations for pharmacists regarding the prescribing of drugs from frequently identified ATC groups in this study.

## Figures and Tables

**Table 1:** The total number of all pharmaceutical prescriptions in Poland in the 21-month period—from 1 May 2020 to 31 January 2022—by type of prescription written: pharmaceutical prescription, *pro auctore* prescription, and *pro familiae* prescription.

Frequency Table			
Category	Count	Percentage	Cumulative Percentage
*Pro auctore*	391,884	13.09	13.09
*Pro familia*	415,587	13.88	26.96
Pharmaceutical prescription	2,187,275	73.04	100.00
Missing	0	0.00	100.00

**Table 2:** Analysis of the types of pharmaceutical prescriptions in Poland in each year of the period studied, divided into pharmaceutical prescriptions, *pro auctore* prescriptions, and *pro familiae* prescriptions.

(Chi^2^ = 10,189.68; df = 4; *p* < 0.00001.)—Summary Crosstabulation Frequency Table				
Year	*Pro auctore*	*Pro familiae*	Pharmaceutical Prescription	Row Totals
**2020**	98,115	115,464	702,476	916,055
Column %	25.04%	27.78%	32.12%	
Row %	10.71%	12.60%	76.68%	
Total %	3.28%	3.86%	23.46%	30.59%
**2021**	267,937	274,611	1,370,445	1,912,993
Column %	68.37%	66.08%	62.66%	
Row %	14.01%	14.36%	71.64%	
Total %	8.95%	9.17%	45.76%	63.88%
**2022**	25,832	25,512	114,354	165,698
Column %	6.59%	6.14%	5.23%	
Row %	15.59%	15.40%	69.01%	
Total %	0.86%	0.85%	3.82%	5.53%
**Totals**	391,884	415,587	2,187,275	2,994,746
Total %	13.09%	13.88%	73.04%	100.00%

**Table 3:** The total number of all pharmaceutical prescriptions in Poland in the period of 21 months—from 1 May 2020 to 31 January 2022—taking into account the share of prescriptions with reimbursement.

Frequency Table			
Category	Count	Percentage	Cumulative Percentage
Prescription with reimbursement	96,308	3.22	3.22
Prescription with full payment	2,898,438	96.78	100.00
Missing	0	0.00	100.00

**Table 4:** Analysis of pharmaceutical prescriptions in Poland by year with changes in the share of prescriptions with reimbursement.

(Chi^2^ = 360.6443; df = 2; *p* < 0.00001.)—Summary Crosstabulation Frequency Table			
Year	Prescription with Reimbursement	Prescription with Full Payment	Row
**2020**	31,901	884,154	916,055
Column %	33.12%	30.50%	
Row %	3.48%	96.52%	
Total %	1.07%	29.52%	30.59%
**2021**	59,804	1,853,189	1,912,993
Column %	62.10%	63.94%	
Row %	3.13%	96.87%	
Total %	2.00%	61.88%	63.88%
**2022**	4603	161,095	165,698
Column %	4.78%	5.56%	
Row %	2.78%	97.22%	
Total %	0.15%	5.38%	5.53%
Totals	96,308	2,898,438	2,994,746
Total %	3.22%	96.78%	100.00%

**Table 5:** Analysis of pharmaceutical prescriptions in Poland in the years 2020, 2021, and 2022, including changes in the share of prescriptions with reimbursement in the group of *pro auctore* and *pro familiae* prescriptions.

(Chi^2^ = 614.2365; df = 2; *p* < 0.00001.): “Pro auctore” + “Pro familiae”—Summary Crosstabulation Frequency Table			
Year	Prescription with Reimbursement	Prescription with Full Payment	Row
**2020**	9055	89,060	98,115
Column %	30.78%	24.57%	
Row %	9.23%	90.77%	
Total %	2.31%	22.73%	25.04%
**2021**	18,870	249,067	267,937
Column %	64.15%	68.71%	
Row %	7.04%	92.96%	
Total %	4.82%	63.56%	68.37%
**2022**	1491	24,341	25,832
Column %	5.07%	6.72%	
Row %	5.77%	94.23%	
Total %	0.38%	6.21%	6.59%
Totals	29,416	362,468	391,884
Total %	7.51%	92.49%	100.00%

**Table 6:** Forms of pharmaceutical prescription written throughout the analysis period.

Frequency Table			
Category	Count	Percentage	Cumulative Percentage
Prescription in electronic form	2,976,453	99.39	99.39
Prescription in paper form	18,293	0.61	100.00
Missing	0	0.00	100.00

**Table 7:** Analysis of types of medicines prescribed by a pharmacist according to the Anatomical Therapeutic Chemical Classification (ATC) System.

Frequency Table			
Category ATC	Count	Percentage	Cumulative Percentage
C	749,799	25.04	25.04
N	261,257	8.72	33.76
R	259,824	8.68	42.44
G	210,231	7.02	49.46
D	223,404	7.46	56.92
A	387,746	12.95	69.86
J	250,811	8.38	78.24
M	259,473	8.66	86.90
B	93,637	3.13	90.03
L	3541	0.12	90.15
H	110,528	3.69	93.84
S	119,565	3.99	97.83
P	26,277	0.88	98.71
V	2725	0.09	98.80
*	1	0.00	98.80
Magistral preparation	35,927	1.20	100.00
Missing	0	0.00	100.00

A—alimentary tract and metabolism; B—blood and blood-forming organs; C—cardiovascular system; D—dermatologicals; G—genito-urinary system and sex hormones; H—systemic hormonal preparations, excluding sex hormones and insulins; J—anti-infectives for systemic use; L—antineoplastic and immunomodulating agents; M—musculoskeletal system; N—nervous system; P—antiparasitic products, insecticides, and repellents; R—respiratory system; S—sensory organs; V—various; *—unidentified record.

**Table 8:** Characteristics of pharmacists performing pharmaceutical prescribing in Poland by gender and age of pharmacist.

(Chi^2^ = 12,089.90; df = 2; *p* < 0.00001.)—Summary Crosstabulation Frequency Table			
Age	Sex—Woman	Sex—Man	Row
**Age ≤ 40**	865,656	310,710	1,176,366
Column %	37.80%	44.35%	
Row %	73.59%	26.41%	
Total %	28.94%	10.39%	39.33%
**40 < Age ≤ 60**	1,078,575	310,342	1,388,917
Column %	47.09%	44.30%	
Row %	77.66%	22.34%	
Total %	36.06%	10.38%	46.44%
**Age > 60**	346,058	79,518	425,576
Column %	15.11%	11.35%	
Row %	81.32%	18.68%	
Total %	11.57%	2.66%	14.23%
Totals	2,290,289	700,570	2,990,859
Total %	76.58%	23.42%	100.00%

## Data Availability

All data are available from the corresponding author. Requests may be sent to the corresponding author.

## Supplementary Materials

The following supporting information can be downloaded at: https://www.mdpi.com/article/10.3390/healthcare11243106/s1, Table S1. Types of prescriptions in pharmaceutical prescribing in Poland that fall into the category of independent prescribing. Table S2. Impact of pharmaceutical prescribing in Poland taking into account the division of prescriptions into pharmaceutical prescription, *pro auctore,* and *pro familiae,* on the method of prescription writing (Electronic, Paper). Table S3. Number of prescriptions issued by pharmacists in Poland in the analyzed period by voivodeship. Table S4. List of the most frequently prescribed drug substances by pharmacists by ATC anatomical-therapeutic-chemical classification group code, the prescriptions of which accounted for more than 1% of all drugs prescribed during the 21-month period. Table S5. Age analysis taking into account the gender of the pharmacist. Table S6. Characteristics of pharmaceutical prescribing carried out with full payment and with reimbursement taking into account the age of the pharmacist. Table S7. Characteristics of pharmaceutical prescribing by share of *pro auctore* and *pro familiae* prescriptions vs. age of pharmacist. Figure S1. Pearson’s linear correlation between the number of pharmacists in a voivodeship and the number of pharmaceutical prescriptions. Figure S2. Correspondence Analysis (CA) for variables: Type of pharmaceutical prescription vs. ATC main group. 2D Plot of Row and Column Coordinates-Dimension 1 vs. Dimension 2. Figure S3. Basic descriptive statistics with histogram calculated for the variable: age of pharmacist who performed pharmaceutical prescribing.

## References

[1] Hoti K., Sunderland B., Hughes J., Parsons R. (2010). An evaluation of Australian pharmacist’s attitudes on expanding their prescribing role. Pharm. World Sci..

[2] Lambert M., Smit C.C.H., De Vos S., Benko R., Llor C., Paget W.J., Briant K., Pont L., Van Dijk L., Taxis K. (2022). A systematic literature review and meta-analysis of community pharmacist-led interventions to optimise the use of antibiotics. Br. J. Clin. Pharmacol..

[3] Ogilvie M., Nissen L., Kyle G., Hale A. (2022). An evaluation of a collaborative pharmacist prescribing model compared to the usual medical prescribing model in the emergency department. Res. Social. Adm. Pharm..

[4] Cope L.C., Abuzour A.S., Tully M.P. (2016). Nonmedical prescribing: Where are we now?. Ther. Adv. Drug Saf..

[5] Adams A.J., Weaver K.K. (2016). The Continuum of Pharmacist Prescriptive Authority. Ann. Pharmacother..

[6] Canadian Pharmacists Association (2016). Pharmacists’ Expanded Scope of Practice.

[7] Cope L.C., Tully M.P., Hall J. (2020). An exploration of the perceptions of non-medical prescribers, regarding their self-efficacy when prescribing, and their willingness to take responsibility for prescribing decisions. Res. Social. Adm. Pharm..

[8] Tonna A., Stewart D., McCaig D. (2008). An international overview of some pharmacist prescribing models. J. Malta Coll. Pharm. Pract..

[9] Courtenay M., Gillespie D., Lim R. (2017). Patterns of dispensed non-medical prescribing for antibiotics in primary care across England: A retrospective analysis. J. Antimicrob. Chemother..

[10] Dawoud D., Griffiths P., Maben J., Goodyer L., Greene R. (2011). Pharmacist supplementary prescribing: A step toward more independence?. Res. Social. Adm. Pharm..

[11] Elnour A.A., Raja N.S., Abdi F., Mostafiz F., Elmubarak R.I., Khalil A.M., Hait K.A., Alqahtani M.M., Dabbagh N., Abdulnasser Z. (2022). Protocol for systematic review and meta-analysis of randomized controlled trials, cost-benefit analysis and interrupted time-series interventions on pharmacist’s prescribing. Pharm. Pract..

[12] Nissen L. (2011). Pharmacist prescribing: What are the next steps?. Am. J. Health Syst. Pharm..

[13] Miszewska J., Wrzosek N., Zimmermann A. (2022). Extended Prescribing Roles for Pharmacists in Poland-A Survey Study. Int. J. Environ. Res. Public Health.

[14] The Polish Pharmaceutical Law Act of 6 September 2001. (JL No. 126, Item 1381) Consolidated Text of 15 March 2019 (JL Item 499) and Consolidated Text of 28 May 2021 (JL Item 974). https://isap.sejm.gov.pl/isap.nsf/DocDetails.xsp?id=WDU20011261381.

[15] Act of 10 December 2020 on the Profession of Pharmacist. https://isap.sejm.gov.pl/isap.nsf/download.xsp/WDU20210000097/O/D20210097.pdf.

[16] Gancarz K. Pharmacist Prescribing in Poland, European Pharmacists Professsional Forum 11 October 2022. https://www.nia.org.pl/2022/10/12/polscy-farmaceuci-liderami-zmian-w-europie-spotkanie-w-ramach-prac-pgeu-za-nami.

[17] Central Statistical Office of Poland (2022). Small Statistical Yearbook of Poland, Warsaw. https://stat.gov.pl/obszary-tematyczne/roczniki-statystyczne/roczniki-statystyczne/maly-rocznik-statystyczny-polski-2022,1,24.html.

[18] Central Statistical Office of Poland. https://stat.gov.pl/files/gfx/portalinformacyjny/pl/defaultaktualnosci/5468/6/31/1/ludnosc_stan_i_struktura_oraz_ruch_naturalny_w_przekroju_terytorialnym_na_31-12-2021.pdf.

[19] Stewart D.C., George J., Bond C.M., Diack H.L., McCaig D.J., Cunningham S. (2009). Views of pharmacist prescribers, physicians and patients on pharmacist prescribing implementation. Int. J. Pharm. Pract..

[20] Raghunandan R., Howard K., Marra C.A., Tordoff J., Smith A. (2021). Identifying Community Pharmacist Preferences For Prescribing Services in Primary Care in New Zealand: A Discrete Choice Experiment. Appl. Health Econ. Health Policy.

[21] Courtenay M., Gerada C., Haywood J. (2011). Working with non-medical prescribers. Br. J. Gen. Pract..

[22] Pharmaceutical Group of the European Union (2019). Pharmacy 2030: A Vision for Community Pharmacy in Europe. https://www.pgeu.eu/wp-content/uploads/2019/04/Pharmacy-2030_-A-Vision-for-Community-Pharmacy-in-Europe.pdf.

[23] Johnson C.F., Maskrey M., MacBride-Stewart S. (2022). New ways of working releasing general practitioner capacity with pharmacy prescribing support: A cost-consequence analysis. Fam. Pract..

[24] Dolovich L., Pottie K., Kaczorowski J., Farrell B., Austin Z., Rodriguez C., Gaebel K., Sellors C. (2008). Integrating family medicine and pharmacy to advance primary care therapeutics. Clin. Pharmacol. Ther..

[25] Kroezen M., van Dijk L., Groenewegen P.P., Francke A.L. (2011). Nurse prescribing of medicines in Western European and Anglo-Saxon countries: A systematic review of the literature. BMC Health Serv. Res..

[26] Law M.R., Ma T., Fisher J., Sketris I.S. (2012). Independent pharmacist prescribing in Canada. Can. Pharm. J. (Ott.).

[27] Dolovich L., Austin Z., Waite N., Chang F., Farrell B., Grindrod K., Houle S., McCarthy L., MacCallum L., Sproule B. (2018). Pharmacy in the 21st century: Enhancing the impact of the profession of pharmacy on people’s lives in the context of health care trends, evidence and policies. Can. Pharm. J. (Ott.).

[28] Alghamdi S.S.A., Hodson K., Deslandes P., Gillespie D., Haines K., Hulme E., Courtenay M., Deslandes R. (2020). Prescribing trends over time by non-medical independent prescribers in primary care settings across Wales (2011–2018): A secondary database analysis. BMJ Open.

[29] Deslandes P., Blowers H., Haines K., Hodson K., Deslandes R. (2022). Medicines prescribed by non-medical independent prescribers in primary care in Wales: A 10-year longitudinal study April 2011-March 2021. BMJ Open.

[30] Central Statistical Office of Poland. https://stat.gov.pl/obszary-tematyczne/zdrowie/zdrowie/apteki-i-punkty-apteczne-w-2021-roku,15,6.html.

[31] Central Statistical Office of Poland. https://www.oecd-ilibrary.org/sites/d6227663-en/index.html?itemId=/content/component/d6227663-en.

[32] Central Statistical Office of Poland Pharmacies and Pharmacy Points in 2021. https://stat.gov.pl/obszary-tematyczne/zdrowie/zdrowie/zdrowie-i-ochrona-zdrowia-w-2020-roku,1,11.html.

[33] Association of Pharmacists of Employers of Polish Pharmacies Pharmacies in Poland-Report. http://aptekarze.org.pl/wp-content/uploads/2019/05/zappa_raport_2019_19_04_2019_dr.pdf.

[34] Pharmacist in Poland (2019). Nationwide Image Research. Warsaw. https://www.nia.org.pl/wp-content/uploads/2019/04/Raport_Farmaceuta_w_Polsce_2019.pdf.

[35] Zimmermann A., Płaczek J., Wrzosek N., Owczarek A. (2021). Assessment of Pharmacists Prescribing Practices in Poland—A Descriptive Study. Healthcare.

[36] World Health Organization (1994). The Role of the Pharmacist in the Health Care System. https://apps.who.int/iris/bitstream/handle/10665/63817/WHO_PHARM_97_599.pdf?sequence=1&isAllowed=y.

[37] Polish Ministry of Health Pharmaceutical Care Report. Comprehensive Analysis of the Implementation Process. https://www.gov.pl/web/zdrowie/opieka-farmaceutyczna---raport.

[38] Pharmaceutical Group of the European Union (2021). Position Paper on the Role of Community Pharmacists in COVID-19—Lessons Learned from the Pandemic 2 Position Paper on the Role of Community Pharmacists in COVID-19—Lessons Learned from the Pandemic. https://www.pgeu.eu/wp-content/uploads/2020/03/PGEU-Position-Paper-on-on-the-Lessons-Learned-from-COVID-19-ONLINE.pdf.

[39] Baqir W., Miller D., Richardson G. (2012). A brief history of pharmacist prescribing in the UK. Eur. J. Hosp. Pharm. Sci..

[40] Hughes C.A., Makowsky M., Sadowski C.A., Schindel T.J., Yuksel N., Guirguis L.M. (2014). What prescribing means to pharmacists: A qualitative exploration of practising pharmacists in Alberta. Int. J. Pharm. Pract..

[41] Kay O.C., Bajorek B.V., Brien J. (2006). Pharmacist Prescribing Activities—An Electronic Survey on the Opinions of Australian Pharmacists. J. Pharm. Pract. Res..

[42] Hale A.R., Coombes I.D., Stokes J., McDougall D., Whitfield K., Maycock E., Nissen L. (2013). Perioperative medication management: Expanding the role of the preadmission clinic pharmacist in a single centre, randomised controlled trial of collaborative prescribing. BMJ Open.

[43] Marotti S.B., Kerridge R.K., Grimer M.D. (2011). A Randomized Controlled Trial of Pharmacist Medication Histories and Supplementary Prescribing on Medication Errors in Postoperative Medications. Anaesth. Intensive Care.

[44] Jebara T., Cunningham S., MacLure K., Awaisu A., Pallivalapila A., Stewart D. (2018). Stakeholders’ views and experiences of pharmacist prescribing: A systematic review. Br. J. Clin. Pharmacol..

[45] Schindel T.J., Yuksel N., Breault R., Daniels J., Varnhagen S., Hughes C.A. (2017). Perceptions of pharmacists’ roles in the era of expanding scopes of practice. Res. Soc. Adm. Pharm..

[46] Polish Ministry of Health, The State Medicines Policy 2018–2022. https://www.gov.pl/web/premier/polityka-lekowa-panstwa-2018-2022.

