# Supplementary material for: Assessment of the Prescribing Pharmacist’s Role in Supporting Access to Prescription-Only Medicines—Metadata Analysis in Poland
Source: Healthcare (Basel). 2023 Dec 5;11(24):3106. doi: 10.3390/healthcare11243106 (PMC10743265; doi:10.3390/healthcare11243106)
Supplement: Supplementary file 1 [file healthcare-11-03106-s001.zip › healthcare-2638194-supplementary.pdf]

Supplementary Information

Assessment of prescribing pharmacists role in supporting the access to prescription-only medicines – metadata analysis in Poland

Table S1. Types of prescriptions in pharmaceutical prescribing in Poland that fall into the category of independent prescribing.

	**Prescription pro auctore/ pro familiae**	**Pharmaceutical prescription**
**Pharmacist authorized**	any pharmacist with a license to practice	pharmacist in a community pharmacy
**Eligible patient**	pharmacist or pharmacist's family member (to the extent indicated in the law)	each patient
**How to issue a prescription**	in electronic form using the gabinet.gov.pl application, paper prescription	pharmacy system, in exceptional situations paper prescription
**Amount of drug prescribed Type of drug**	- in the case of an e-prescription for a maximum of 180 days of use according to the dosage indicated on the prescription<br>- in the case of a paper prescription, for a maximum of 120 days of use according to the dosage indicated on the prescription	- in the case of e-prescription for a maximum of 180 days of use according to the dosage indicated on the prescription<br>- in the case of a paper prescription for a maximum of 120 days of use according to the dosage indicated on the prescription
**Type of drug**	medicinal products with prescription availability category (Rx), excluding narcotic and psychotropic substances	in the case of a paper prescription for a maximum of 120 days of use according to the dosage indicated on the prescription
**Drug payment**	for full payment or with reimbursement	for full payment
**Prescription records**	issuing pharmacist	pharmacy
**Reason for issuance**	- entered only in the pharmacist's records<br>- according to medical knowledge and the pharmacist's diagnosis	- placed on the prescription<br>- risk to patient's health and life

Table S2. Impact of pharmaceutical prescribing in Poland taking into account the division of prescriptions into pharmaceutical prescription, *pro auctore,* and *pro familiae,* on the method of prescription writing (Electronic, Paper).

Tab S1 (Chi^2 =37853.91; df=2; p < 0.00001.) - Summary Crosstabulation Frequency Table			
**Year**	**Electronic**	**Paper**	**Row**
**Pro auctore**	382524	9360	391884
Column %	*12,85%*	*51,17%*	
Row %	*97,61%*	*2,39%*	
Total %	*12,77%*	*0,31%*	*13,09%*
**Pro familiae**	408638	6949	415587
Column %	*13,73%*	*37,99%*	
Row %	*98,33%*	*1,67%*	
Total %	*13,65%*	*0,23%*	*13,88%*
**Pharmaceutical prescription**	2185291	1984	2187275
Column %	*73,42%*	*10,85%*	
Row %	*99,91%*	*0,09%*	
Total %	*72,97%*	*0,07%*	*73,04%*
Totals	2976453	18293	2994746
Total %	*99,39%*	*0,61%*	*100,00%*

Table S3. Number of prescriptions issued by pharmacists in Poland in the analyzed period by voivodeship.

Tab S2 - Frequency table			
**Voivodeship***	**Count**	**Percent**	**Cumulative Percentage**
Lubusz (lubuskie)	64743	2,16	2,16
Opole (opolskie)	39557	1,32	3,48
Lesser Poland (małopolskie)	407584	13,61	17,09
Lodz (łódzkie)	197315	6,59	23,68
Lublin (lubelskie)	184018	6,14	29,83
Greater Poland (wielkopolskie)	484409	16,18	46,00
Swietokrzyskie (świętokrzyskie)	149556	4,99	51,00
Silesia (śląskie)	395019	13,19	64,19
Masovia (mazowieckie)	310164	10,36	74,54
Podlaskie (podlaskie)	100338	3,35	77,89
Pomerania (pomorskie)	116881	3,90	81,80
Lower Silesian (dolnośląskie)	200345	6,69	88,49
West Pomerania (zachodniopomorskie)	82253	2,75	91,23

Kuyavia-Pomerania(kujawsko-pomorskie)	80920	2,70	93,93
Subcarpathia (podkarpackie)	136607	4,56	98,50
Warmia-Masuria (warmińsko-mazurskie)	44209	1,48	99,97
Missing	828	0,03	100,00
Missing	0	0,00	100,00

* voivodeship code numer on figures 5 and 6: Lubusz (lubuskie) 04; Opole (opolskie) 08; Lesser Poland (małopolskie) 06; Lodz (łódzkie) 05; Lublin (lubelskie) 03; Greater Poland (wielkopolskie) 15; Swietokrzyskie (świętokrzyskie) 13; Silesia (śląskie) 12; Masovia (mazowieckie) 07; Podlaskie (podlaskie) 10; Pomerania (pomorskie) 11; Lower Silesian (dolnośląskie) 01; West Pomerania (zachodniopomorskie) 16; Kuyavia-Pomerania(kujawsko-pomorskie) 02; Subcarpathia (podkarpackie) 09; Warmia-Masuria (warmińsko-mazurskie) 14.

Table S4. List of the most frequently prescribed drug substances by pharmacists by ATC anatomical-therapeutic-chemical classification group code, the prescriptions of which accounted for more than 1% of all drugs prescribed during the 21-month period.

**Tab S3 - Frequency table**		
**Category**	**Count**	**Percent**
C07AB07	94742	3,16
H03AA01	89709	3,00
C07AB02	53617	1,79
C09AA05	50811	1,70
M01AB05	48975	1,64
A10BA02	46256	1,54
J01FA10	44081	1,47
A02BC02	44034	1,47
Missing	35927	1,20
G03AA12	33752	1,13
C07AB12	33110	1,11
C10AA07	32887	1,10
S01CA06	31194	1,04

Table S5. Age analysis taking into account the gender of the pharmacist.

**Tab S4**								
**Descriptive Statistics (Age)**								
**Variable**	**Valid N**	**Mean**	**Confidence: SR-95% CI**	**Confidence: SR+95% CI**	**Median**	**Minimum**	**Maximum**	**Std.Dev.**
Age_(All)	2990859	45,50	45,485	45,513	44,0	25,0	88,0	12,30
Age_(Woman)	2290289	46,15	46,131	46,164	44,0	25,0	88,0	12,54
Age_(Man)	700570	43,38	43,352	43,405	42,0	25,0	87,0	11,23

Table S6. Characteristics of pharmaceutical prescribing carried out with full payment and with reimbursement taking into account the age of the pharmacist.

Tab 14 (Chi^2 =1307.375; df=2; p < 0.00001.) - Summary Crosstabulation Frequency Table			
Age	**Prescription with reimbursement**	**Prescription with full payment**	**Row**
Age ≤ 40	43141	1133225	1176366
Column %	*44,89%*	*39,15%*	
Row %	*3,67%*	*96,33%*	
Total %	*1,44%*	*37,89%*	*39,33%*
40 < Age ≤ 60	41001	1347916	1388917
Column %	*42,66%*	*46,56%*	
Row %	*2,95%*	*97,05%*	
Total %	*1,37%*	*45,07%*	*46,44%*
Age > 60	11958	413618	425576
Column %	*12,44%*	*14,29%*	
Row %	*2,81%*	*97,19%*	
Total %	*0,40%*	*13,83%*	*14,23%*
Totals	96100	2894759	2990859
Total %	*3,21%*	*96,79%*	*100,00%*

Table S7. Characteristics of pharmaceutical prescribing by share of *pro auctore* and *pro familiae* prescriptions *vs.* age of pharmacist.

Tab S6 (Chi^2 = 136799.7; df=4; p < 0.00001.) - Summary Crosstabulation Frequency Table				
**Age**	**Pro auctore**	**Pro familiae**	**Pharmaceutical prescription**	**Row Totals**
**Age ≤ 40**	207086	238488	730792	1176366
Column %	*52,89%*	*57,46%*	*33,46%*	
Row %	*17,60%*	*20,27%*	*62,12%*	
Total %	*6,92%*	*7,97%*	*24,43%*	*39,33%*
**40 < Age ≤ 60**	156691	153476	1078750	1388917
Column %	*40,02%*	*36,98%*	*49,39%*	
Row %	*11,28%*	*11,05%*	*77,67%*	
Total %	*5,24%*	*5,13%*	*36,07%*	*46,44%*
**Age > 60**	27766	23077	374733	425576
Column %	*7,09%*	*5,56%*	*17,16%*	
Row %	*6,52%*	*5,42%*	*88,05%*	
Total %	*0,93%*	*0,77%*	*12,53%*	*14,23%*
**Totals**	391543	415041	2184275	2990859
Total %	*13,09%*	*13,88%*	*73,03%*	*100,00%*

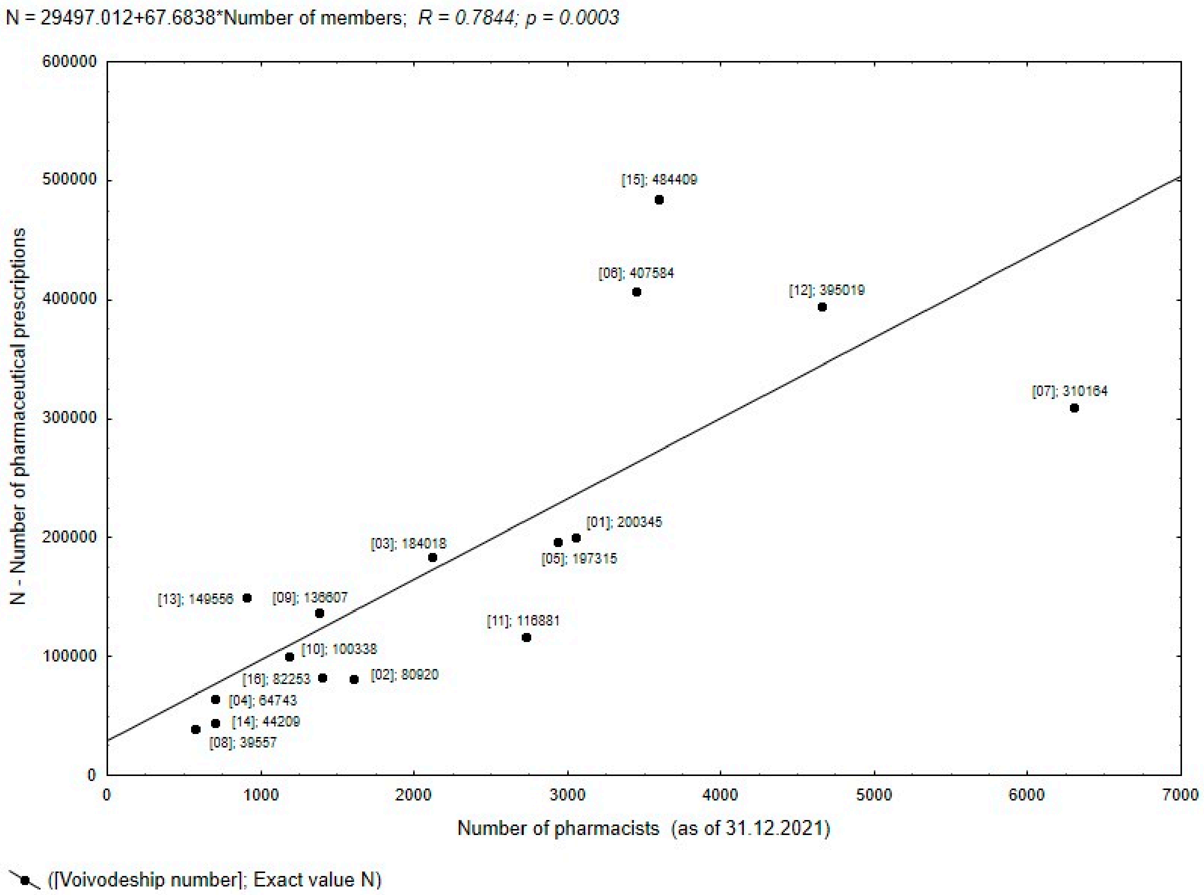

Figure S1. Pearson's linear correlation between the number of pharmacists in a voivodeship and the number of pharmaceutical prescriptions.

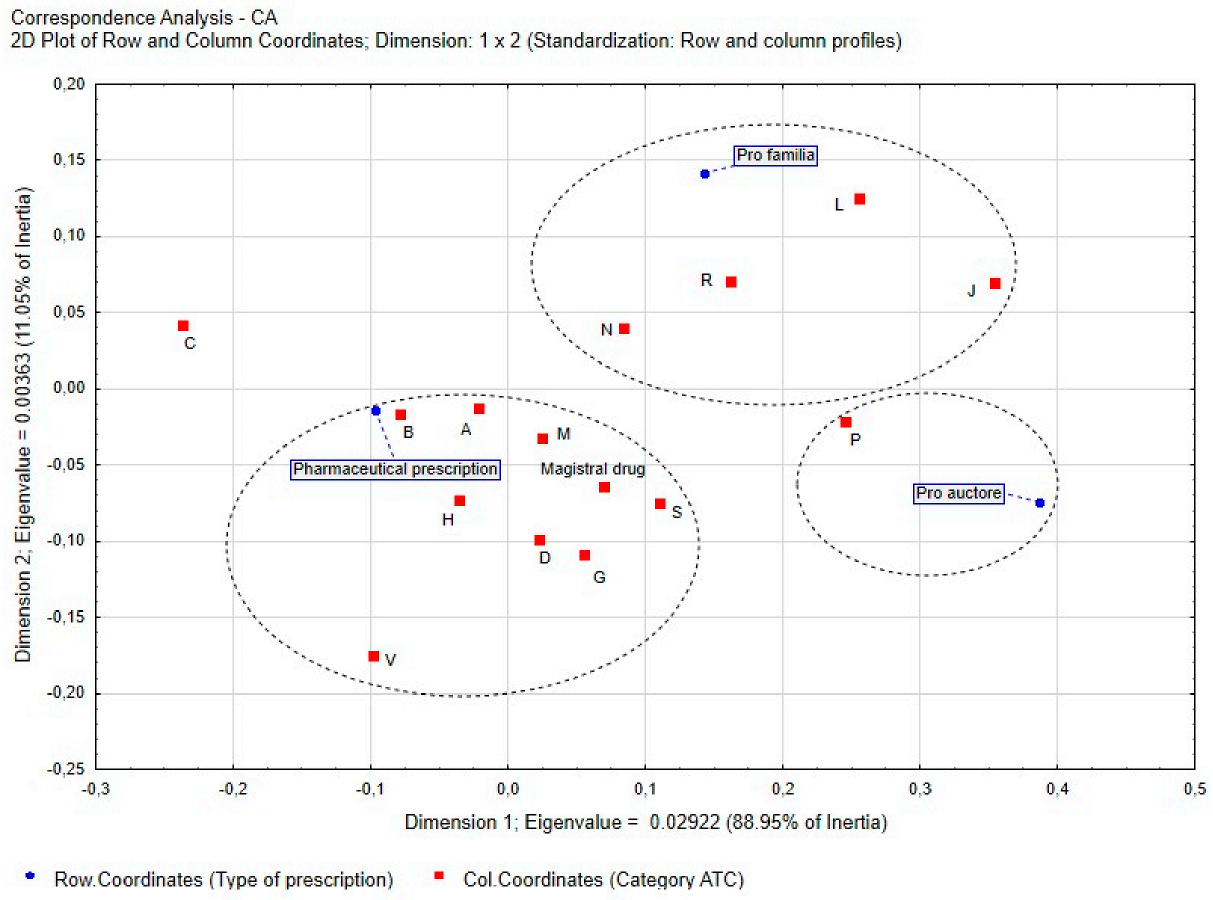

Figure S2. Correspondence Analysis (CA) for variables: Type of pharmaceutical prescription vs. ATC main group. 2D Plot of Row and Column Coordinates - *Dimension 1 vs. Dimension 2.*

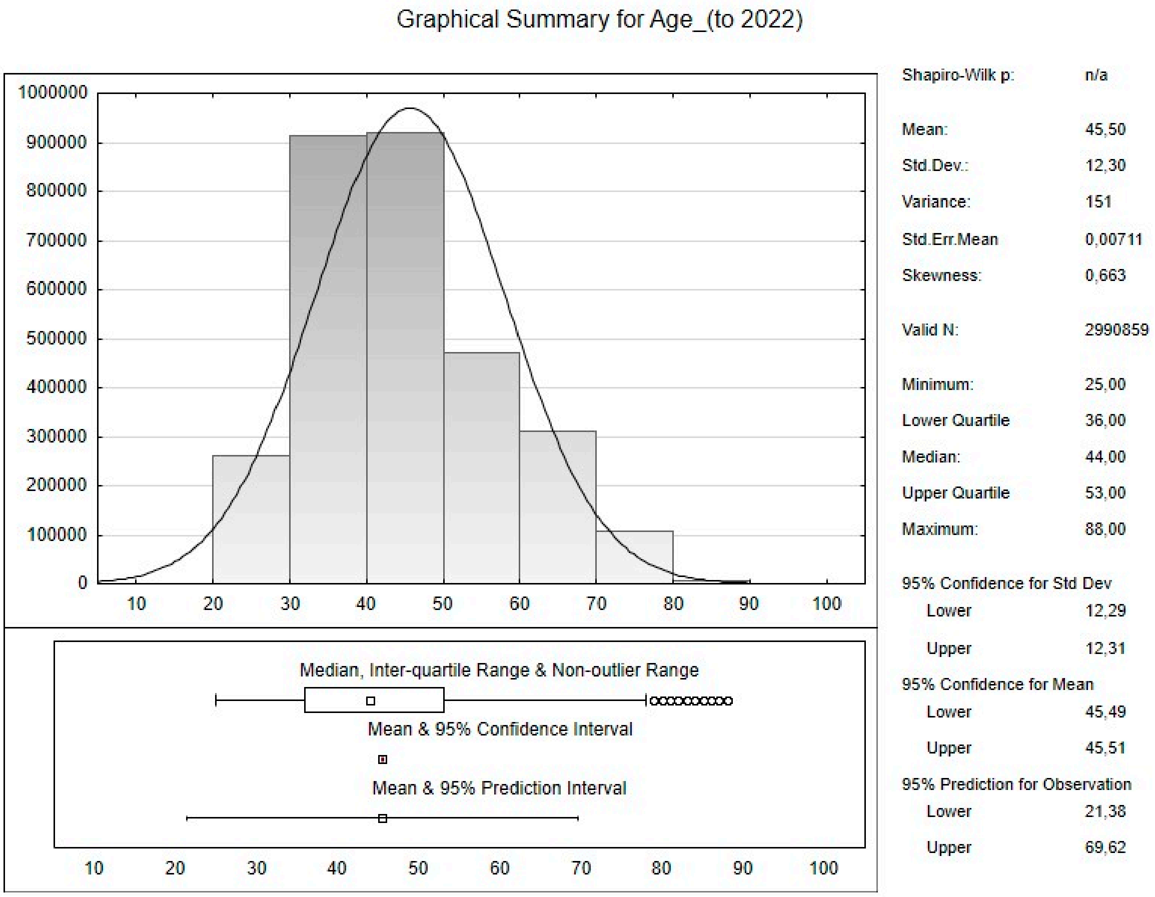

Figure S3. Basic descriptive statistics with histogram calculated for the variable: age of pharmacist who performed pharmaceutical prescribing.
